# Supplementary material for: Comprehensive transcriptomic analysis of cell lines as models of primary tumors across 22 tumor types
Source: Nat Commun. 2019 Aug 8;10:3574. doi: 10.1038/s41467-019-11415-2 (PMC6687785; doi:10.1038/s41467-019-11415-2)
Supplement: Supplementary file 1 — Supplementary Information [file 41467_2019_11415_MOESM1_ESM.pdf]

Comprehensive transcriptomic analysis of cell lines as models of primary samples across 22 tumor types

Yu et al.

Supplementary Table

Supplementary Table 1: Number of differentially expressed genes between primary tumor samples and cell lines

Disease	Genes upregulated in TCGA	Genes upregulated in CCLE	Total DEG
**BLCA**	1088	866	1954
**BRCA**	1076	864	1940
**CHOL**	1002	1112	2114
**COADREAD**	1074	780	1854
**DLBC**	1644	1343	2987
**ESCA**	697	460	1157
**HNSC**	773	532	1305
**GBM**	1621	1401	3022
**KIRC**	1661	1586	3247
**LAML**	755	769	1524
**LGG**	2030	2046	4076
**LIHC**	1829	1772	3601
**LUAD**	1110	884	1994
**LUSC**	1079	716	1795
**MESO**	1144	1116	2260
**OV**	1238	852	2090
**PAAD**	1369	1000	2369
**PRAD**	1299	1343	2642
**SKCM**	790	565	1355
**STAD**	1023	551	1574
**THCA**	1685	1677	3362
**UCEC**	1207	854	2061

Supplementary Figures

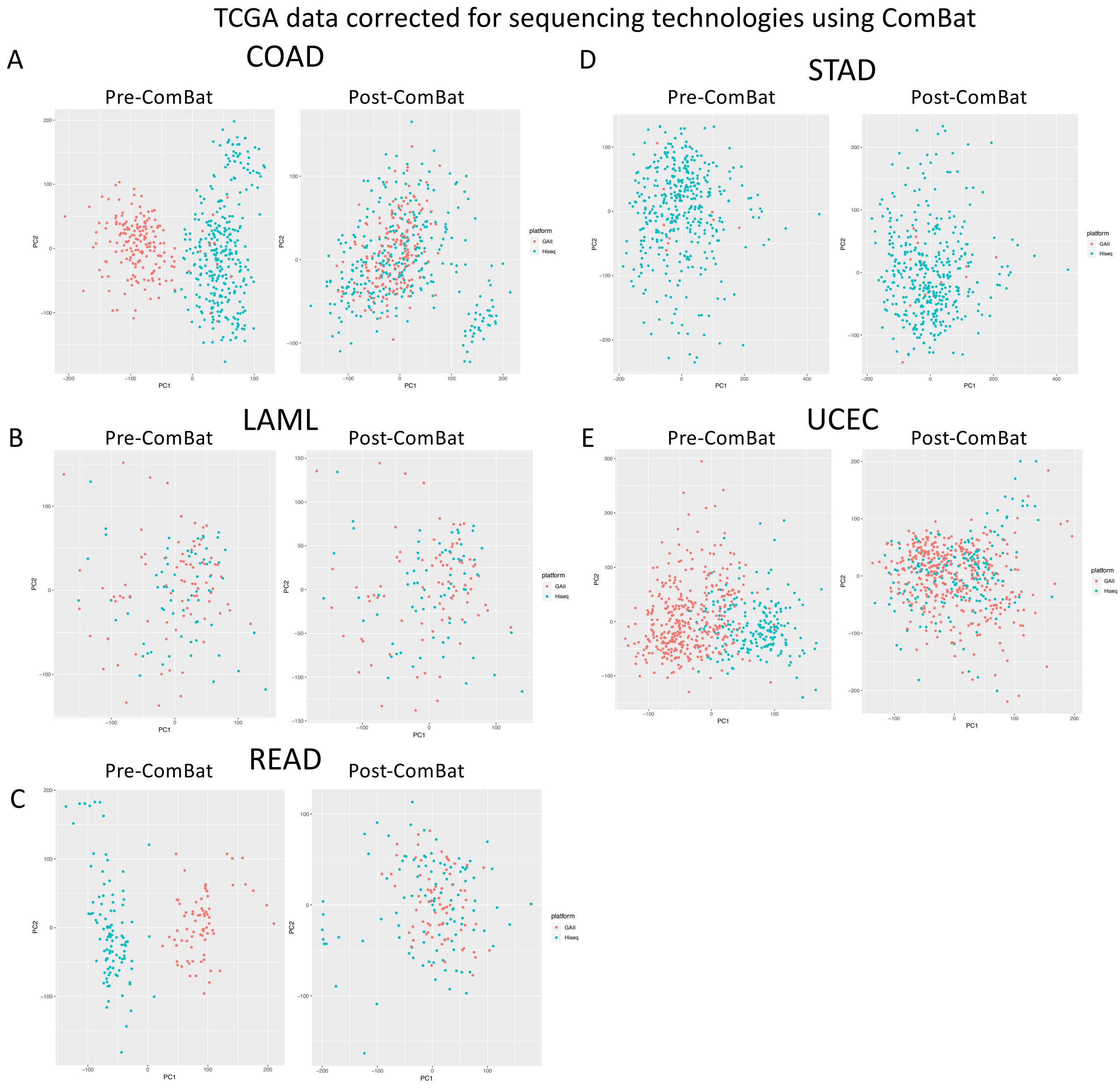

Supplementary Figure 1. Sequencing platform batch effects were corrected for using ComBat in the following tumors types: COAD (A), LAML (B), READ (C), STAD (D), and UCEC (E). Samples sequenced on Illumina's Genome Analyzer II Platform (GAII) are in red and samples sequenced on Illumina's HiSeq platform are in turquoise. The plots on the left shows PC1 and PC2 of the samples before ComBat correction and the plots on the right shows the samples after ComBat correction.

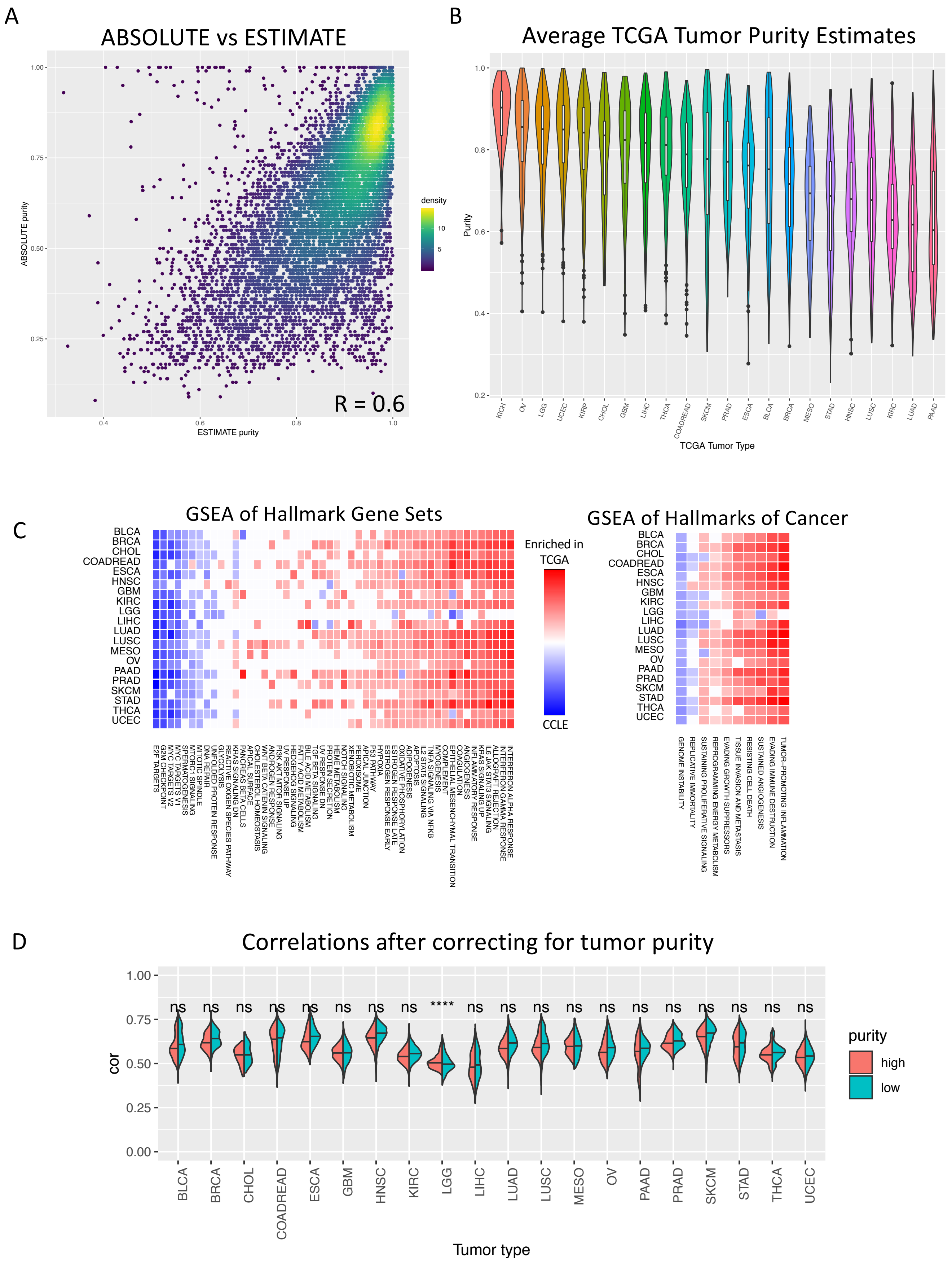
A
ABSOLUTE vs ESTIMATE
ABSOLUTE purity
ESTIMATE purity
density
R = 0.6
B
Average TCGA Tumor Purity Estimates
Purity
TCGA Tumor Type
KICH OV LGG UCEC KIRP CHOL GBM LIHC THCA COADREAD SKCM PRAD ESCA BLCA BRCA MESO STAD HNSC LUSC KIRC LUAD PAAD
C
GSEA of Hallmark Gene Sets
GSEA of Hallmarks of Cancer
Enriched in
TCGA
CCLE
BLCA BRCA CHOL COADREAD ESCA HNSC GBM KIRC LGG LIHC LUAD LUSC MESO OV PAAD PRAD SKCM STAD THCA UCEC
GENOME INSTABILITY
REPLICATIVE IMMORTALITY
SUSTAINING PROLIFERATIVE SIGNALING
REPROGRAMMING ENERGY METABOLISM
EVADING GROWTH SUPPRESSORS
TISSUE INVASION AND METASTASIS
RESISTING CELL DEATH
SUSTAINED ANGIOGENESIS
EVADING IMMUNE DESTRUCTION
TUMOR-PROMOTING INFLAMMATION
D
Correlations after correcting for tumor purity
cor
Tumor type
purity
high
low
ns
****
BLCA BRCA CHOL COADREAD ESCA GBM HNSC KIRC LGG LIHC LUAD LUSC MESO OV PAAD PRAD SKCM STAD THCA UCEC

Supplementary Figure 2. Confounding effect of tumor purity on GSEA and correlation analysis. A. Purity estimates calculated using ESTIMATE (x-axis) and ABSOLUTE (y-axis) are highly correlated (R = 0.6, p-value < 2.2e-16). B. Violin plots showing the purity estimates of the primary tumors separated by tumor type. C. (left) Gene Set Enrichment Analysis (GSEA) of differential expression results without purity as a covariate between primary tumor samples and cell lines in hallmark gene sets from MSigDB. NES are shown for pathways with FDR < 5%. Before adjusting for tumor purity, immune related pathways are strongly enriched in primary tumor samples. (right) Gene Set Enrichment Analysis (GSEA) of differential expression results without purity as a covariate between primary tumor samples and cell lines in hallmarks of cancer pathways. NES are shown for pathways with FDR < 5%. D. After adjusting for tumor purity, correlations between cell lines and high purity primary tumor samples (red) are significantly higher than correlations between cell lines and low purity primary tumor samples (turquoise) in only 1/20 tumor types using the one-sided Wilcoxon rank sum test. P-values are indicated by symbols above the violin plots with ns corresponding to p-value > 0.05 and four stars corresponding to p-value <= 0.0001.

A

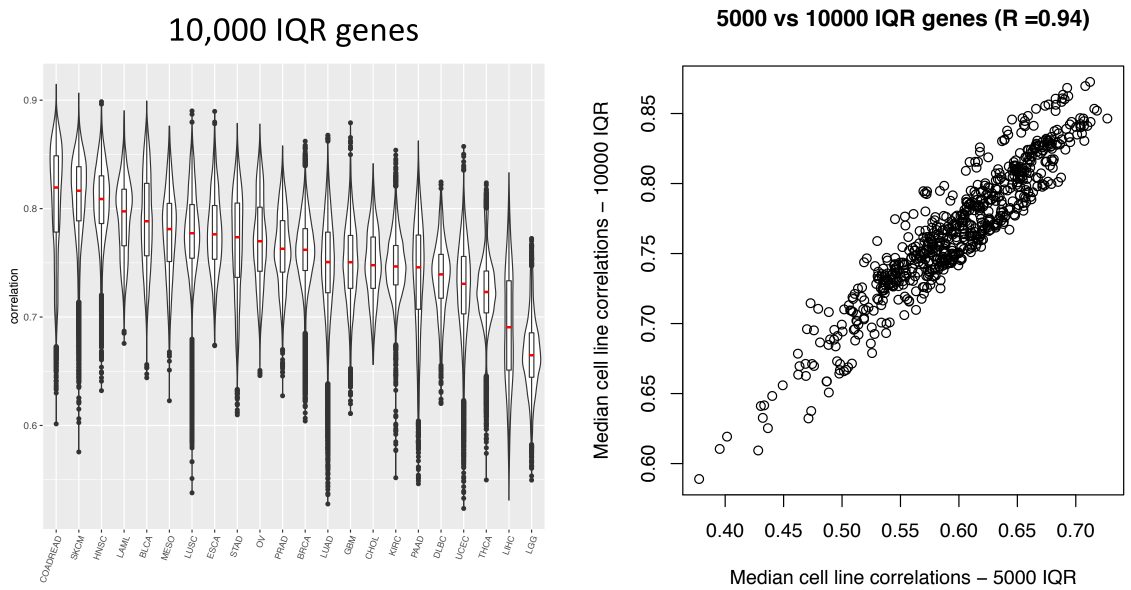

B

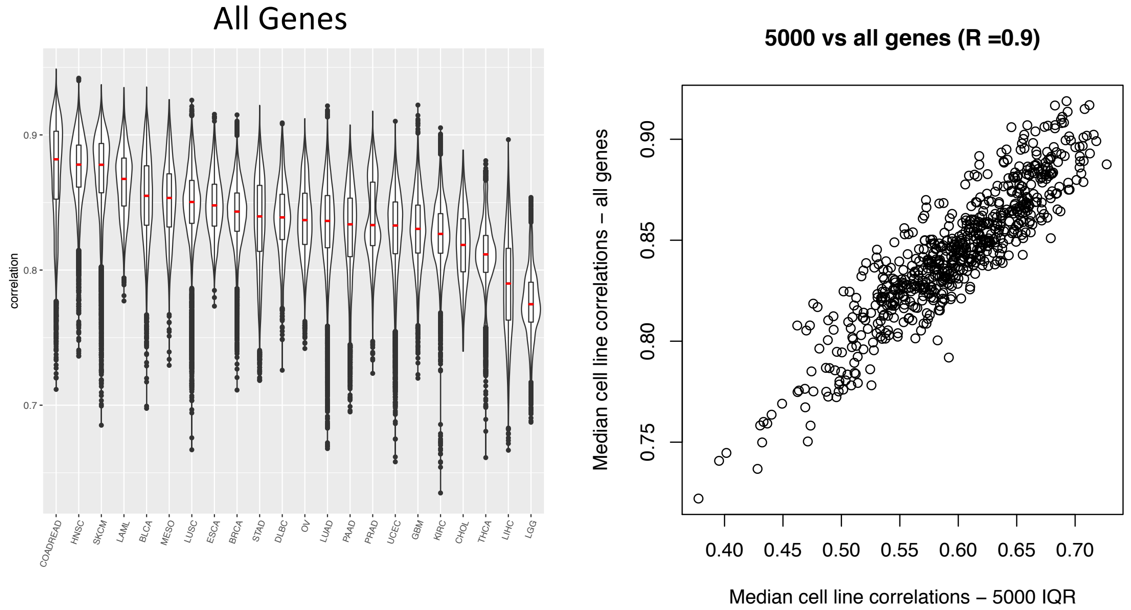

Supplementary Figure 3. Varying the number of genes used in the correlation analysis does not significantly affect the results. A. Correlations calculated using 10,000 most variable genes (left) separated by tumor type do not differ significantly from correlations calculated using 5,000 most variable genes. The median correlation coefficients of each cell line compared to their primary tumor samples (right) are highly correlated when using 5,000 IQR genes (x-axis) to 10,000 IQR genes (y-axis) (Pearson correlation = 0.94, p-value < 2.2e-16). B. Correlations calculated using all genes (left) do not differ significantly from correlations calculated using 5,000 most variable genes. The median correlation coefficients of each cell line compared to their primary tumor samples (right) are highly correlated when using 5,000 IQR genes (x-axis) to all genes (y-axis) (Pearson correlation = 0.90, p-value < 2.2e-16).

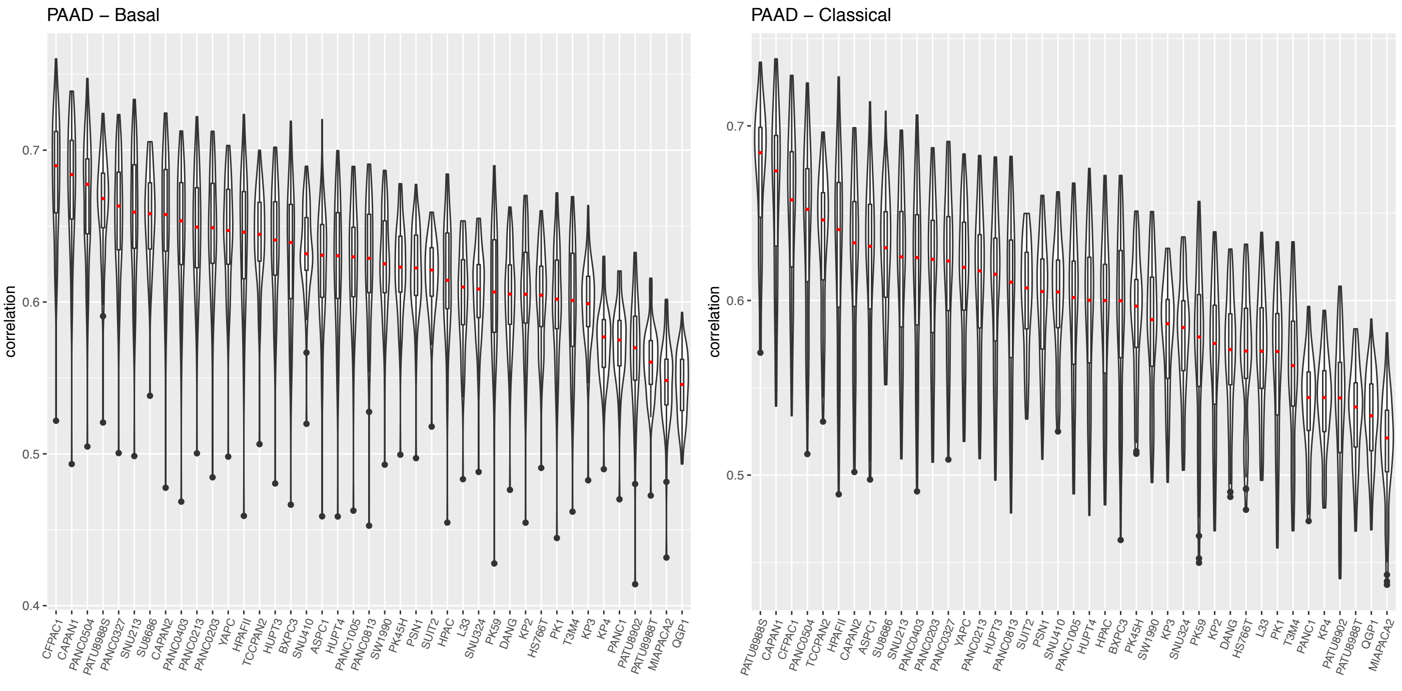

Supplementary Figure 4. Spearman's correlations between PAAD cell lines and PAAD basal primary tumors (left) and PAAD classical primary tumors (right) using the 5,000 most variable genes. The correlations are separated by cell lines (x-axis). In the overlaid boxplot, the red center line displays the median, the box limits display the upper and lower quartiles, and the whiskers display 1.5 times the interquartile range.
