# Supplementary material for: Comprehensive transcriptomic analysis of cell lines as models of primary tumors across 22 tumor types
Source: Nat Commun. 2019 Aug 8;10:3574. doi: 10.1038/s41467-019-11415-2 (PMC6687785; doi:10.1038/s41467-019-11415-2)
Supplement: Supplementary file 3 — Description of Additional Supplementary Files [file 41467_2019_11415_MOESM3_ESM.pdf]

Description of Additional Supplementary Files

Supplementary Data 1: File contains the barcodes of the TCGA tumors used in the study

Supplementary Data 2: File contains the names of the CCLE cell lines used in the study

Supplementary Data 3: File contains the top 500 most variable genes by interquartile range for each tumor type. The GO analysis for these genes are provided in the subsequent sheets.

Supplementary Data 4: File contains the full pair-wise correlation matrix of CCLE cell lines compared to TCGA tumors across all tumor types.

Supplementary Data 5: File contains the ovarian cell line rankings from the Domcke et al. publication compared to the expression-based rankings derived from this study.

Supplementary Data 6: File contains the subtype predictions for 9 tumor types. Each sheet contains cell line subtype predictions, the p-value and FDR for each prediction, and the expression-based ranking of the cell line compared to the primary tumors in each subtype.

Supplementary Data 7: File contains a list of the TCGA-110 cell lines, their median correlations with their respective primary tumors, their predicted subtype if available, and their tumor type.
